# The Prevalence of Amnestic and Non-Amnestic Mild Cognitive Impairment and Its Association with Different Lifestyle Factors in a South Italian Elderly Population

**DOI:** 10.3390/ijerph19053097

**Published:** 2022-03-06

**Authors:** Alessandro Oronzo Caffò, Giuseppina Spano, Luigi Tinella, Antonella Lopez, Elisabetta Ricciardi, Fabrizio Stasolla, Andrea Bosco

**Affiliations:** 1 Department of Educational Sciences, Psychology, Communication, University of Studies of Bari, 70122 Bari, Italy; giuseppina.spano@uniba.it (G.S.); luigi.tinella@uniba.it (L.T.); antonella.lopez@uniba.it (A.L.); elisabetta.ricciardi@uniba.it (E.R.); andrea.bosco@uniba.it (A.B.); 2 Law Department, “Giustino Fortunato” University of Benevento, 82100 Benevento, Italy; f.stasolla@unifortunato.eu

**Keywords:** aging, mild cognitive impairment, risk factors, protective factors, prevalence

## Abstract

Mild Cognitive Impairment (MCI) is a transition stage between normal aging and dementia and can be useful to monitor the cognitive status of people at risk of dementias. Our aims were to investigate the prevalence of amnestic and non-amnestic MCI in a South Italian elderly population, and to identify socio-demographic, clinical and lifestyle factors associated with MCI. A cross-sectional retrospective population study on 839 community-dwelling participants over 60 years of age was carried out. Elderly people were administered a brief neuropsychological screening to identify their cognitive and functional status, and a questionnaire to investigate several socio-demographic, clinical, and lifestyle factors. Prevalence estimate for MCI was 12.0% (95% CI: 10.0–14.5%), for amnestic MCI was 7.4% (95% CI: 5.8–9.4%), and for non-amnestic MCI was 4.6% (95% CI: 3.4–6.4%), for people older than 60 years of age. Logistic regression models, corrected for age, sex, and education, revealed a significant association of MCI with the following factors: age, education, intellectual activities, and topographical disorientation. On the other hand, education, clinical factors (e.g., depression level and perceived physical pain), lifestyle factors (e.g., smoking, alcohol, and leisure/productive activities), dietary habits, quality of life, and self-reported topographical disorientation were non-significantly associated with MCI. Prevalence estimates and the association of MCI and its subtypes with risk and protective factors were discussed in comparison with the most recent systematic reviews and meta-analyses.

## 1. Introduction

Mild Cognitive Impairment (MCI) is known as a transitional stage between normal aging and dementia. It has been defined as a condition of objective cognitive impairment with no dementia. MCI involves the onset and evolution of cognitive impairments beyond those expected based on an individual’s age and education, but which are not significant enough to interfere with her or his daily activities [1,2,3]. Following Petersen’s MCI classification, it is possible to distinguish four subtypes, amnestic MCI single domain ((aMCIsd); memory impaired only), amnestic MCI multiple domain ((aMCImd) memory impaired plus one or more other cognitive domains), nonamnestic MCI single domain ((naMCIsd); impairment in one nonmemory domain), and nonamnestic MCI multiple domain ((naMCImd); impairment in two or more nonmemory domains) [1,2]. These subtypes show differences in clinical outcomes. Both aMCIsd and aMCImd conditions are more likely to convert to Alzheimer’s disease (AD), with remarkable differences in progression rates among them. Non-aMCI conditions are instead more likely to convert in other types of dementia, such as vascular dementia, frontotemporal dementia, or dementia with Lewy body [4].

Several studies investigated prevalence of MCI all over the world. Mitchell and Shiri-Feshki [4] found that MCI cases ranged from 2% to 30% in a normal population and from 6% to 85% (with an average value of 40%) in a clinical setting. Ward and colleagues [5] reported a prevalence of MCI between 3% and 42% with a median value of 26.4% and a prevalence of aMCI between 0.5% and 31.9% with a median value of 4.9%. Alexander and colleagues [6] found that estimates of prevalence for European populations were comprised between 2.5% and 14.9% for MCI, and between 4.9% and 8.7% for aMCI. Hu and colleagues [7] found a higher MCI prevalence in studies conducted in the community (25%, 95% CI: 18–33%) with respect to those conducted in clinical contexts (20%, 95% CI: 14–25%). This result is not surprising, since, as authors state, clinical samples are more likely to meet criteria for advanced disease states than participants from the community. In confirmation of this, the dementia rate (DR) and Alzheimer’s disease rate (AR) were found to be higher in the clinical sample (DR: 39%, 95% CI: 31–47%; AR: 35%, 95% CI: 27–43%) than in the community (DR: 25%, 95% CI: 17–32%; AR: 19%, 95% CI: 14–24%). More recently, Pessoa and colleagues [8] reported an MCI prevalence ranging from 0.5% to 41.8% with a pooled estimate equal to 17.3% (95% CI: 13.8–20.8%), and Parnetti and colleagues [9] found an estimate of preclinical AD equal to 22% (95% CI: 18–26%). Subsequent updates on estimates for the Chinese elderly population [10] reported a differentiated MCI estimates based on age group, as follows: 8% (95% CI: 6–10.1%) in individuals 60–69 years of age, 13.1% (95% CI: 10.6–15.6%) in individuals 70–79 years of age, and 23.4% (95% CI: 18.3–28.6%) when 80 years of age or older. This systematic review revealed a MCI prevalence on the general elderly population equal to 14% (95% CI: 12–17%). Similarly, a meta-analysis [11] on MCI prevalence on the Chinese population 55 years of age or older indicated estimates ranging from 14.8% (95% CI: 12.2–17.6%) to 21.2% (95% CI: 17.5–25.2%) depending on the different diagnostic criteria used. In reference to the same population, Lu et al. [12] reported a MCI prevalence of 12.2% (95% CI: 10.6–14.2%), also specifying a aMCI prevalence of 10.9% (95% CI: 7.7–15.4%). A wide range of MCI prevalence can be also found in a meta-analysis on derived data from nine countries, i.e., Brazil, Mexico, Argentina, Colombia, Peru, Cuba, Dominican Republic, Venezuela, and Costa Rica [13]; the pooled prevalence of all-type MCI across the included studies revealed a prevalence of 14.95% (95% CI: 6.81–25.52%) and a prevalence of aMCI estimated of 6.30% (95% CI: 3.09–10.54%). However, a wide gap among estimates can be observed by stratifying by age and education (i.e., prevalence ranging from 6.8% to 25.5% and between 3.1% and 10.5%, for MCI and aMCI, respectively).

Prevalence studies were conducted also in the Italian elderly population. Ten studies have been recognized, showing a degree of variability similar to that found in the aforementioned systematic reviews [14,15,16,17,18,19,20,21,22,23]. Indeed, MCI prevalence estimates varied from 3.2% [15] to 24.5% [20] with a median value equal to 6.1%. Most of such studies were conducted in Northern Italy and few studies used a multicenter sample. To the best of our knowledge, no study has been conducted exclusively in Southern Italy. Moreover, few studies investigated the prevalence of MCI subtypes, at least regarding the presence of memory impairment (aMCI and naMCI) [16,18,19,20,22].

Several studies also investigated the association between cognitive decline and dementia and a series of protective and risk factors. Recent systematic reviews [24,25,26,27] highlighted the role of these modifiable predictors in order to reduce MCI and dementia outcomes. A non-exhaustive list of potential modifiable predictors includes the following factors: years of formal education, physical activity, Mediterranean diet, cognitive training, moderate alcohol consumption, social engagement, traumatic brain injury, mid-life obesity, metabolic syndrome, current smoking, caffeine, fatty acids, diabetes, history of depression, sleep disturbances, and hyperlipidemia.

Another factor which is of interest in MCI as well as in an established condition of dementia is topographical disorientation (TD), which is defined by the seminal work by Aguirre and D’Esposito [28] as a particular condition which determines the loss of spatial orientation and is characterized by the difficulty in acquiring spatial information in new and unknown environments, and in encompassing familiar environments such as one’s neighborhoods or one’s house. TD and spatial memory impairments occur relatively early as an effect of cognitive decline in aging, and it is possible to observe transient episodes of TD, other than in people suffering from dementia, especially of Alzheimer’s type, even in prodromal stages of dementia, namely MCI [29]. Following Aguirre and D’Esposito taxonomy, TD is not a unitary concept, but it is possible to split it into four components: egocentric disorientation, heading/allocentric disorientation, landmark agnosia, and anterograde disorientation. Recent literature showed that familiarity with the environment might represent a protective factor from spatial memory impairment in non-pathological cognitive aging, in particular for allocentric tasks. Indeed, information consolidated across a huge number of retrieval episodes seems to be solidly preserved in elderly people, making them to obtain a performance comparable with that of younger people [30,31,32].

The aims of the present study are (a) to investigate the prevalence of participants with MCI and its subtypes, namely amnestic and non-amnestic MCI, in a South Italian elderly population, (b) to identify socio-demographic, clinical and lifestyle protective and risk factors associated with MCI and its subtypes.

## 2. Methods

### 2.1. Participants

**Figure 1:**
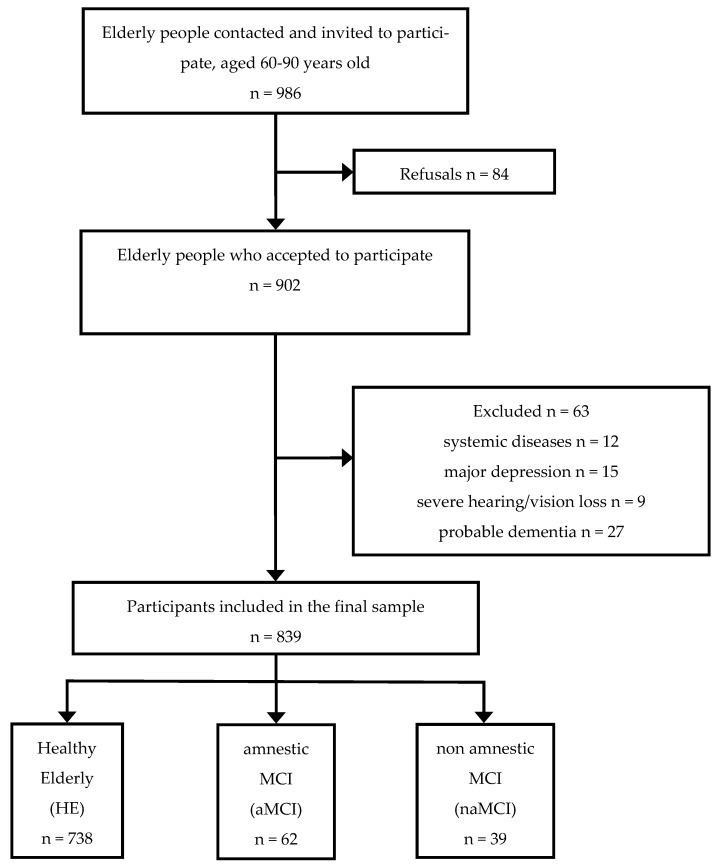
Flow diagram showing the enrollment process.

Nine hundred eighty-six elderly people from the metropolitan area of Bari, Italy, were contacted and invited to participate in the study. All of them were volunteers recruited from senior centers and third age universities with the support of a proxy informant, generally undergraduate or graduate students, trainees, employers of the centers and general practitioners. They were consecutively contacted and eventually enrolled between October 2016 and May 2018. Inclusion criteria for study participation were: (a) higher than 60 years of age (b) being native Italian speaker, (c) residence in a region of Southern Italy (i.e., Abruzzo, Basilicata, Calabria, Campania, Molise, Apulia, Sicily, and Sardinia), (d) having normal or corrected-to-normal vision, (e) not having a history of suspected uncompensated systemic/traumatic/psychiatric diseases, or with severe vision/hearing loss, which can affect cognition. Eighty-four elderly people refused to take part in the study. Nine hundred and two accepted to participate. Following the administration of the anamnestic and neuropsychological part of the protocol (see paragraph Materials and Procedure for details), sixty-three participants were excluded since they had uncompensated systemic diseases (n = 12), major depression (n = 15), severe hearing/vision loss (n = 9), or probable dementia (n = 27). Eight hundred thirty-nine participants (467 women) were included in the final sample. Figure 1 shows the enrollment process. All participants were blinded to the hypothesis of the study and signed a consent form for participating. The Ethical Committee of the Institution approved the study protocol and the whole study was performed following Helsinki Declaration and its later amendments.

### 2.2. Materials and Procedure

Elderly participants were administered (a) a general anamnesis, carried out by supervised trainees in psychogeriatric care assessment, in order to exclude people with a history of suspected uncompensated systemic/traumatic/psychiatric diseases, or with severe vision/hearing loss, which can affect cognition, and (b) a standardized neuropsychological battery, in order to establish a diagnosis of probable MCI and its subtypes (amnestic and non-amnestic MCI), according to the MCI working group of the European Consortium on AD [33]. In particular, the neuropsychological assessment consisted of the following validated tests and scales:

(i) Global cognitive function was evaluated by the Montreal Cognitive Assessment (MoCA) [34], which recently demonstrated to predict the preservation of daily functional activities, such as driving, in older people [35]. This test showed good reliability (Cronbach’s α = 0.83) [34] while the best cut-off used in an Italian sample for discriminating participants with probable cognitive impairment was demonstrated elsewhere and it was 17 [34];
(ii) A possible occurrence of functional decline, usually associated with a severe cognitive impairment and with dementia was evaluated by the Activities of Daily Living (ADL) and Instrumental Activities of Daily Living (IADL) [36]. Both these scales demonstrated moderate to good interclass correlation coefficients (i.e., r = 0.70–0.91) [37,38];
(iii) The 15-item version of the Geriatric Depression Scale (GDS) [39], which is a reliable (Cronbach’s α = 0.91) [40] measure of depression in older people, was administered in order to exclude major depressive symptoms. In this scale, scores less than 5 are considered normal, scores between 5 and 11 suggest mild/moderate depression, and scores between 12 and 15 indicate severe depression [41];
(iv) Subjective complaints regarding memory loss, which demonstrate to be associated to age-related frailty conditions such as falls, low mood, and impaired executive abilities [42,43], were evaluated by the Subjective Memory Complaints questionnaire (SMCq) [44]. The questionnaire demonstrated good reliability (Cronbach’s α = 0.86) [44]) and adequate screening accuracy for MCI, dementia, and cognitive disorders (i.e., range: 60.1–94.6%) [45];
(v) Verbal episodic memory was evaluated by the Rey Auditory Verbal Learning Test [46], with both immediate (cut-off: 28.53; [47] and delayed recall (cut-off: 4.69; [47]) which showed ana alpha reliability equal to 0.84;
(vi) Executive function was evaluated by the Frontal assessment Battery (FAB) [48] and the Clock Drawing Test (CDT) [49], by considering 13.5 and 6.55 as cut-off scores, respectively [50]. The FAB showed good values of inter-rater reliability (r = 0.96), test-retest reliability (r = 0.85), and internal consistency (Cronbach’s α = 0.73) [51]. The CDT showed an interclass correlation coefficient ranging from 0.84 to 0.93 depending by the scoring method [52].

Moreover, each participant was administered an ad-hoc protocol including either single-item questions or validated scales in order to gather information and/or measure the following socio-demographic, clinical, and lifestyle factors: age, sex, education, depression level, familiarity with AD, traumatic brain injuries (TBIs), perceived physical pain, smoking, alcohol, waist-hip ratio (WHR), body mass index (BMI), second language, physical activities, cultural activities, intellectual activities, productive activities, social activities, quality of life, physical health, mental health, sleep quality, topographical disorientation self-report [53], recent newly learned spatial information in an egocentric and allocentric format [30,54], food group 1 (carbohydrates), food group 2 (cured and smoked meats), food group 3 (white meat), food group 4 (red meat), food group 5 (milk), food group 6 (dairy products and cheeses), food group 7 (eggs), food group 8 (fish meat), food group 9 (raw and cooked vegetables with leaves), food group 10 (vegetables and legumes), food group 11 (fruit), water daily consumption, carbonated drinks daily consumption, beer daily consumption, and wine daily consumption. All of the factors were based on a self-report measure, except for the Ego-Allo task (EAT) [54], which was composed of two subtasks, and was to assess egocentric and allocentric spatial memory based on recent and newly learned spatial information, in a table-top format. Participants were instructed to memorize the position and the characteristics of three three-dimensional solids (shape and color). In the testing phase solids were presented on the table, in order to judge distances between them and the observer (egocentric judgment), or between the solids themselves (allocentric judgment). The maximum total score was 8 points for egocentric and 8 points for allocentric judgment, respectively. Table 1 reports the socio-demographic, clinical and lifestyle factors investigated and their related measure.

The entire procedure was made clear to the participants beforehand. Participants were assessed individually in a well-lit and quiet room without disturbances. Data were collected in one session. The whole assessment lasted a maximum of 2 h. The order of the tasks was the same mentioned in the text. Breaks were allowed upon request.

### 2.3. Statistical Analysis

Data were analyzed using R 3.5.1 statistical software [55]. We obtained demographic data (sex, age, and years of education) and scores on neuropsychological, clinical and lifestyle measures. Normality of distribution was assessed for all considered variables. Following a series of Shapiro–Wilk tests, all variables significantly deviated from normality, with at least *p* < 0.01. Since such test is biased for large sample size, skewness and kurtosis indexes were also calculated. Except for ADL, IADL, RAVLT delayed recall, and WHR, skewness and kurtosis did not exceed the critical threshold [56]. A Bonferroni correction for multiple comparisons was applied, taking into account the following parameters: the initial alpha level (0.05), the number of tests (39), the mean correlation between predictors (0.21). Following such procedure, a one-tailed value of *p* < 0.012 was determined to be statistically significant. Pearson’s chi-squared analysis was performed to assess for differences in the distribution of sex among the four groups. A series of univariate analysis of variance (ANOVAs) were carried out to compare means from the three diagnostic groups for demographic continuous data, and scores on neuropsychological tests used to establish a diagnosis of probable MCI and its subtypes. The significant ANOVAs were further explored using post hoc Tukey HSD for unequal N. This test is a generalization of the Tukey’s test to the case of unequal sample sizes.

Prevalence estimates were calculated as the number of elderly people with MCI divided by the number of elderly people included in the final sample. Confidence intervals were calculated using the formula of confidence intervals for sample proportions.

A series of logistic regressions were performed in order to estimate the probability, expressed in terms of odds ratio, of having a diagnosis of cognitive impairment following to the presence of one factor. A second series of logistic regressions were performed in order to avoid confounding effects by analyzing the association of each factor considered and age, sex, and education level.

## 3. Results

Means, standard deviations, frequencies, F or χ^2^ values, P levels for statistics for demographic variables, for functional and neuropsychological tests, as well as Tukey HSD for unequal N post hoc tests are reported in Table 2. Means, standard deviations, frequencies, F or χ^2^ values, P levels for statistics for all the predictors included in the logistic regression models, as well as Tukey HSD for unequal N post hoc tests are reported in Supplementary Materials (Table S1). Correlations between the employed variables were also analyzed. Age was found negatively correlated with education, perceived physical pain, activities (physical, social, cultural, productive), both physical and mental health, and the performance on the EAT. Significant and positive associations were found between depression, age, and topographical disorientation measures, as well as between education and second language, physical, cultural, intellectual activities, egocentric, allocentric and total score at the EAT. The male sex was significantly and positively associated with smoking, alcohol consumption, and WHR. Despite this, the male sex was associated with higher self-reported physical and mental health. Moreover, the perceived physical pain showed to be positively associated with alcohol consumption, WHR, and second language, and negatively with self-reported topographical disorientation. Finally, the physical activity positively correlated with both physical and mental health, and with the egocentric, allocentric, and the total score at the EAT.

Seven hundred and thirty-eight participants (403 women) were classified as healthy elderly (HE), and 101 (64 women) as participants with probable Mild Cognitive Impairment (MCI): the latter group was further classified as participants with probable amnestic MCI ((aMCI) 62 persons, 39 women) and with probable non amnestic MCI ((naMCI) 39 persons, 25 women). The mean scores of neuropsychological measures for each group were compatible with Italian normative data. HE were significantly younger and had significantly lower scores at SMCq than aMCI and naMCI, which in turn did not differ between them. Moreover, HE were significantly more educated and had significantly higher scores at MoCA than aMCI and naMCI, which in turn did not differ between them. The aMCI group obtained significantly lower scores at the RAVLT immediate and delayed recall than HE and naMCI, which in turn did not differ between them. The naMCI group had significantly lower scores at FAB and CDT compared with aMCI and HE, which in turn did not differ between them. Finally, there were no differences between the three groups for sex distribution, ADL, IADL, and GDS scores.

Prevalence estimate for MCI was 12.0% (95% CI: 10.0, 14.5%). Prevalence estimate for aMCI was 7.4% (95% CI: 5.8, 9.4%), and for naMCI was 4.6% (95% CI: 3.4, 6.4%).

The first series of logistic regressions revealed significant associations between the group of MCI participants and the following factors (see Table 3): age (OR: 1.08; 95% CI: 1.04, 1.11, *p* < 0.001), education (OR: 0.84; 95% CI: 0.79, 0.89, *p* < 0.001), perceived physical pain (OR: 1.32; 95% CI: 1.07, 1.59, *p* < 0.01), physical (OR: 0.81; 95% CI: 0.69, 0.93, *p* < 0.01), cultural (OR: 0.70; 95% CI: 0.54, 0.89, *p* < 0.01), intellectual (OR: 0.76; 95% CI: 0.68, 0.84, *p* < 0.001), quality of life related to physical health (OR: 0.91; 95% CI: 0.85, 0.97, *p* < 0.01), egocentric (OR: 1.20; 95% CI: 1.09, 1.32, *p* < 0.001) and allocentric (OR: 1.20; 95% CI: 1.09, 1.32, *p* < 0.01) components of topographical disorientation, and the total score of topographical disorientation (OR: 1.13; 95% CI: 1.07, 1.20, *p* < 0.001). After correcting for age, sex, and education, significant associations were found for intellectual activities (OR: 0.83; 95% CI: 0.74, 0.93, *p* < 0.01), egocentric components of topographical disorientation (OR: 1.16; 95% CI: 1.05, 1.29, *p* < 0.01), and the total score of topographical disorientation (OR: 1.10; 95% CI: 1.04, 1.17, *p* < 0.01).

Logistic regressions conducted with the group of aMCI showed a significant association with the following factors (see Table 4): age (OR: 1.09; 95% CI: 1.04, 1.13, *p* < 0.001), education (OR: 0.82; 95% CI: 0.75, 0.89, *p* < 0.001), physical (OR: 0.75; 95% CI: 0.59, 0.91, *p* < 0.01), and intellectual (OR: 0.73; 95% CI: 0.63, 0.83, *p* < 0.001) activities, quality of life related to physical health (OR: 0.90; 95% CI: 0.83, 0.98, *p* < 0.01), egocentric (OR: 1.32; 95% CI: 1.17, 1.48, *p* < 0.001), and allocentric (OR: 1.31; 95% CI: 1.16, 1.47, *p* < 0.001) components of topographical disorientation, as well as the total score of topographical disorientation (OR: 0.84; 95% CI: 0.78, 0.90, *p* < 0.001). After correcting for age, sex, and education, significant associations were found for intellectual activities (OR: 0.80; 95% CI: 0.69, 0.93, *p* < 0.001), egocentric (OR: 1.30; 95% CI: 1.14, 1.48, *p* < 0.001) and allocentric (OR: 1.25; 95% CI: 1.09, 1.42, *p* < 0.001) components of topographical disorientation, and the total score of topographical disorientation (OR: 1.18; 95% CI: 1.09, 1.27, *p* < 0.001).

Logistic regressions conducted with the group of naMCI showed a significant association with the following factors (see Table 5): education (OR: 0.86; 95% CI: 0.78, 0.94, *p* < 0.01) and intellectual activities (OR: 0.80; 95% CI: 0.68, 0.94, *p* < 0.01). After correcting for age, sex, and education, no significant associations were found.

## 4. Discussion

The present study investigated the prevalence of MCI and its subtypes (i.e., amnestic, and non-amnestic MCI) in a South Italian elderly population, as well as the association of those groups with several socio-demographic, clinical and lifestyle factors.

Prevalence estimates seem to be in line with those obtained by previous studies conducted on Italian elderly population. Ten studies investigated prevalence of MCI in the Italian elderly population [14,15,16,17,18,19,20,21,22,23]. Although estimates showed a great variability, ranging from 3.2% to 24.5%, they had an average value of 10.7%. Four studies [16,19,20,21] were conducted in Northern Italy, and showed an average value of 12.0%, with a range from 5.0% to 24.5%. Five studies [15,17,18,22,23] were multicenter, and showed an average value of 9.8%, with a range from 3.2% to 21.6%. One study was conducted in Center Italy and found a prevalence estimate of 6.1%. One study [57] investigated the prevalence of aMCI in the Italian population, and found a value of 4.9%, slightly lower than that obtained in the present study. Several socio-demographic and socio-economic factors, as well as design and methodological issues, might explain the great variability in studies conducted in the same country, and further studies are warranted in order reach a consensus about a reliable estimate of prevalence.

Protective factors associated with MCI and with aMCI were education and intellectual activities. The only significant protective factor associated with naMCI was education.

Regarding education, our results are in line with systematic reviews which found a significant association between education and cognitive decline and dementia [24,58,59]: the more educated an individual is, the less likelihood an individual has of developing cognitive decline and dementia. Furthermore, a recent meta-analysis [60] registered a risk reduction of 8% for AD and 7% for all-type dementia for each year of education. This result can be read in the frame of cognitive reserve hypothesis [61,62], which postulates that cognitive reserve reduces the prevalence and incidence of AD or vascular dementia (VaD). Among those who have greater initial cognitive reserve (in contrast to those with less reserve) greater brain pathology occurs before the clinical symptoms of disease becomes manifest. Thus, clinical disease onset triggers a faster decline in cognition and function, and increased mortality among those with initial greater cognitive reserve [60].

Also, regarding intellectual activities, results of the present investigation seem to converge with those found for people with dementia in a systematic review [63], which showed robust evidence that complex patterns of mental activity in the early-, mid-, and late-life stages were associated with a significant reduction in dementia incidence. In this case, the hypothesis of cognitive and behavioral brain reserve might explain the association, as complex intellectual and mental activities across the lifespan allow flexible cognitive repertoires to be deployed in the face of underlying neural dysfunction [62].

Risk factors associated with MCI were age and topographical disorientation, referred to the egocentric component and to the total score of the test employed. The risk factor associated with aMCI were age and topographical disorientation, referred to both egocentric and allocentric components and to the total score of the test employed. No significant risk factors emerged for the group of naMCI.

Age is obviously known as the first risk factor associated with MCI and dementia, the higher the age the higher the risk of developing cognitive decline due to neurodegenerative processes [3].

Regarding topographical disorientation, it was found to be significant for both MCI and aMCI, but not for naMCI. It is noteworthy that the association was present only with the objective measures of topographical disorientation. This result is consistent with studies which documented the frequency of TD in both AD and MCI. For example, Pai and Jacobs [64] found that 61 of the 112 patients with AD residing in a community in Southern Taiwan presented with TD over the course of the study, 28 had TD at a very early stage of the disease, and 33 developed TD within the next 3 years. Those findings were consistent with those of Hort and colleagues [65] documenting the presence of spatial navigation disorders in amnestic MCI patients (aMCI). The Authors used a human analogue of the Morris water maze task to study egocentric and allocentric navigation in patients with AD, with MCI subtypes, in people with subjective memory complaints and healthy controls. Results showed that AD patients and amnestic MCI multiple domain (aMCImd) group were impaired in all subtests (i.e., egocentric, allocentric, and egocentric/allocentric in both real and virtual versions). Weniger and colleagues [66] compared 29 patients with aMCI with 29 healthy controls on two virtual reality navigation tasks assessing allocentric and egocentric spatial memory. Behavioral results showed that aMCI patients were significantly more impaired than controls in both allocentric and egocentric tasks. Rusconi and colleagues [67] submitted 18 healthy subjects and 18 MCI patients (9 aMCI and 9 naMCI) to a neuropsychological battery and to a new spatial navigation test reproducing an ideal city. They found that aMCI patients performed worse in learning a new route, in replacing landmarks in the city, and in drawing a map of the city, whilst naMCI patients’ performance was not different from that observed in healthy subjects.

Contrary to expectations, other considered factors did not show significant associations with the three cognitively impaired conditions. In this regard, a meta-analysis based on data from 18 longitudinal studies [68] found that depression was associated with a higher risk of dementia, and the use of antidepressants did not seem to be a protective factor of dementia, but a risk factor for MCI. However, it is worth specifying that the aforementioned meta-analysis mostly considered patients with a diagnosed depression, while we opted for a brief screening tool, i.e., the Geriatric Depression Scale (GDS), for excluding major depression. The same can be noted for other well-known reliable protective/risk factors against/favoring developing MCI and dementia, such as, physical activity, smoking, alcohol consumption, and BMI, e.g., [69,70,71,72]. Regarding physical activity, we can speculate that the use of a single item, as in this study, may be insufficiently sensitive in capturing the variability within habits among our study participants. As for other health-related behavior, mixed findings are available; for example, alcohol consumption showed to be a protective factor for dementia, but not for MCI, whilst an opposite pattern can be found for BMI and exercising [73].

In conclusion, the present work offers further information on MCI, aMCI, and naMCI prevalence in an elderly population in Southern Italy. More importantly, it provides an overview on the potential protective/risk role of a considerable number of demographic, clinical, and lifestyle factors not limited to the MCI condition but also considering the aMCI subtype and the far less investigated naMCI subtype condition.

## 5. Limitations

The present study has some limitations, mainly related to the method employed for collecting data about the clinical and lifestyle factors. Most of them were indeed based on the answer of the participant to the questionnaire, and thus were self-reported information. Such answers can be biased by the effect of social desirability, since the participant might have the tendency to answer questions in a manner that will be viewed favorably by others. Another distortion can be produced by the recall bias, since participants might not remember properly previous events or experiences or omit relevant details. Another limitation comes from the fact that some factors were collected on the basis of a single item, which can be an incomplete and reductive measure of a more complex construct. Those weaknesses limit the possibility to draw a full generalization of the results obtained to the whole Italian elderly population. In order to remediate to those limitations, in further research it would be appropriate to collect information by also interviewing a proxy informant, and by employing more valid and reliable measure for each construct investigated.

## 6. Conclusions

It has been demonstrated that two-thirds of people with MCI progress to dementia [74]. This rate is sufficient to justify the effort in detecting the related modifiable risk factors in order to prevent and slow such progression. If we acknowledge, on one hand, that some factors are impossible to prevent (such as, age), on the other hand, there are other risk factors on which we can act in terms of prevention (such as, dietary habits, and lifestyle). In the absence of pharmacological treatments for cognitive impairment in the early stages, prevention through cognitive and social activities remains the only weapon we have to prevent deterioration and to promote a healthy and active aging. Our findings suggest an association between engagement in intellectual abilities and cognitive decline, this might be considered as a starting point for detecting possible modifiable factors against cognitive decline and dementia. Research challenges for this topic mainly concern the definition of risk and protective factors to be monitored and managed. To this aim, long-term longitudinal studies are warranted to disentangle the role of each factor in preventing specific cognitively impaired conditions (e.g., AD, VaD, MCI, and subtypes) and the transition from MCI to AD and other types of dementia.

## Figures and Tables

**Table 1:** Socio-Demographic, clinical and lifestyle factors investigated and their related measure.

Factor	Measure
Age	Years
Sex	Male/Female
Education	Years
Depression level	Geriatric Depression Scale (GDS, 15 item) total score [24]
Familiarity with AD	Yes/no
Traumatic brain injuries (TBIs)	Yes/no
Perceived physical pain	Likert scale 1–5 (not at all–very much)
Smoking	Ever—never
Alcohol	Likert scale 1–5 (never–every day)
Waist-Hip Ratio (WHR)	Waist circumference/Hip circumference
Body Mass Index (BMI)	Weight (in kg)/Height^2^ (in m)
Second language	Yes/no
Physical activities	Likert scale 1–6 (never –every day)
Cultural activities	Likert scale 1–6 (never–every day)
Intellectual activities	Likert scale 1–6 (never–every day)
Productive activities	Likert scale 1–6 (never–every day)
Social activities	Likert scale 1–6 (never–every day)
Quality of life—physical health	SF-12 Health Survey–Physical health (6 item)
Quality of life—mental health	SF-12 Health Survey–Mental health (6 item)
Sleep quality	Likert scale 1–5 (very good–very bad)
TD Self report	Questionnaire on Everyday Navigational Ability (QuENA, 10 item) total score [29]
TD Ego	Ego-Allo task (8 item) egocentric score [30]
TD Allo	Ego-Allo task (8 item) allocentric score [30]
TD Total	Ego-Allo task (16 item) total score [30]
Food group 1 (carbohydrates)	Likert scale 1–5 (many times per day–never)
Food group 2 (cured and smoked meats)	Likert scale 1–5 (many times per day–never)
Food group 3 (white meat)	Likert scale 1–5 (many times per day–never)
Food group 4 (red meat)	Likert scale 1–5 (many times per day–never)
Food group 5 (milk)	Likert scale 1–5 (many times per day–never)
Food group 6 (dairy products and cheeses)	Likert scale 1–5 (many times per day–never)
Food group 7 (eggs)	Likert scale 1–5 (many times per day–never)
Food group 8 (fish meat)	Likert scale 1–5 (many times per day–never)
Food group 9 (raw and cooked vegetables with leaves)	Likert scale 1–5 (many times per day–never)
Food group 10 (vegetables and legumes)	Likert scale 1–5 (many times per day–never)
Food group 11 (fruit)	Likert scale 1–5 (many times per day–never)
Water	Likert scale 1–5 (more than 1 L per day–never)
Carbonated drinks	Likert scale 1–5 (more than 1 L per day–never)
Beer	Likert scale 1–5 (more than 1 L per day–never)
Wine	Likert scale 1–5 (more than 1 L per day–never)

**Table 2:** Socio-demographic characteristics of the participants, mean scores and standard deviations on the neuropsychological screening tests for the elderly groups, and statistical tests for their differences.

	Healthy Elderly (n = 738)	Amnestic MCI (n = 62)	Non-Amnestic MCI (n = 39)	F o χ^2^	*p*	Post Hoc
Age	72.36 ± 6.44	75.87 ± 6.56	75.13 ± 6.68	11.16	<0.001	HE < aMCI = naMCI
Sex (F/M)	403/335	39/23	25/14	2.78	0.25	
Education	9.44 ± 4.78	6.23 ± 3.39	6.77 ± 4.58	18.38	<0.001	HE > aMCI = naMCI
MoCA	23.19 ± 3.06	16.06 ± 0.86	16.18 ± 0.84	267.40	<0.001	HE > aMCI = naMCI
ADL	5.91 ± 0.44	5.61 ± 1.16	5.79 ± 0.97	1.87	0.16	HE = aMCI = naMCI
IADL	7.61 ± 0.88	6.89 ± 1.9	7.36 ± 1.65	1.62	0.21	HE = aMCI = naMCI
GDS	2.74 ± 2.58	3.45 ± 3.28	3.37 ± 2.16	2.88	0.057	HE = aMCI = naMCI
SMCq	2.58 ± 2.29	5.15 ± 2.66	4.54 ± 2.39	22.72	<0.001	HE < aMCI = naMCI
RAVLT Immediate recall	38.5 ± 10.19	28.63 ± 6.66	38.73 ± 6.1	29.02	<0.001	HE = naMCI > aMCI
RAVLT delayed recall	8.84 ± 3.81	5.83 ± 1.88	9.52 ± 1.95	21.35	<0.001	HE = naMCI > aMCI
FAB	13.9 ± 3.01	12.74 ± 2.96	10.81 ± 2.94	39.41	<0.001	HE = aMCI > naMCI
CDT	8.64 ± 1.14	8.08 ± 1.21	6.77 ± 1.07	96.98	<0.001	HE = aMCI > naMCI

Abbreviations: MoCA: Montreal Cognitive Assessment; ADL: Activities of Daily Living; IADL: Instrumental Activities of Daily Living; GDS: Geriatric Depression Scale; SMCq: Subjective Memory Complaints questionnaire; RAVLT: Rey Auditory Verbal Learning Test; FAB: Frontal Assessment Battery; CDT: Clock Drawing Test.

**Table 3:** Odds ratio (95% confidence interval) and related *p*-value for each factor and MCI group.

	Raw OR	95% CI	*p*	Corrected * OR	95% CI	*p*
Age	1.08	1.04, 1.11	<0.001			
Sex	0.70	0.45, 1.06	0.098			
Education	0.84	0.79, 0.89	<0.001			
Depression level	1.09	1.01, 1.17	<0.05	1.03	0.95, 1.11	0.454
Familiarity with AD	1.28	0.64, 2.39	0.455	1.45	0.70, 2.81	0.294
Traumatic brain injuries (TBIs)	0.91	0.34, 2.02	0.824	0.82	0.30, 1.87	0.659
Perceived physical pain	1.32	1.07, 1.59	<0.01	1.07	0.86, 1.32	0.539
Smoking	0.70	0.44, 1.10	0.132	1.07	0.63, 1.81	0.799
Alcohol	0.68	0.46, 1.00	0.057	0.86	0.56, 1.28	0.457
Waist-Hip Ratio (WHR)	0.33	0.04, 2.39	0.291	0.70	0.07, 6.22	0.762
Body Mass Index (BMI)	1.02	0.98, 1.06	0.372	1.01	0.96, 1.05	0.818
Second language	0.50	0.28, 0.86	<0.05	1.22	0.63, 2.28	0.543
Physical activities	0.81	0.69, 0.93	<0.01	0.90	0.76, 1.04	0.165
Cultural activities	0.70	0.54, 0.89	<0.01	0.96	0.73, 1.22	0.732
Intellectual activities	0.76	0.68, 0.84	<0.001	0.83	0.74, 0.93	<0.01
Productive activities	1.02	0.92, 1.15	0.683	1.01	0.90, 1.14	0.851
Social activities	0.96	0.84, 1.11	0.571	1.03	0.88, 1.20	0.746
Quality of life—physical health	0.91	0.85, 0.97	<0.01	0.98	0.91, 1.06	0.579
Quality of life—mental health	0.98	0.93, 1.03	0.420	1.00	0.95, 1.05	0.910
Sleep quality	1.27	1.03, 1.57	<0.05	1.15	0.93, 1.43	0.200
Topographical disorientation self-report	0.98	0.93, 1.03	0.401	0.98	0.93, 1.03	0.466
Topographical disorientation egocentric component	1.20	1.09, 1.32	<0.001	1.16	1.05, 1.29	<0.01
Topographical disorientation allocentric component	1.20	1.09, 1.32	<0.001	1.14	1.03, 1.26	<0.05
Topographical disorientation total	1.13	1.07, 1.20	<0.001	1.10	1.04, 1.17	<0.01
Food group 1 (carbohydrates)	1.06	0.78, 1.45	0.725	1.02	0.74, 1.42	0.902
Food group 2 (cured and smoked meats)	0.98	0.76, 1.28	0.895	1.01	0.77, 1.34	0.924
Food group 3 (white meat)	0.99	0.73, 1.35	0.925	0.87	0.63, 1.22	0.403
Food group 4 (red meat)	1.32	0.97, 1.80	0.078	1.27	0.93, 1.75	0.138
Food group 5 (milk)	0.94	0.81, 1.10	0.422	0.84	0.72, 0.99	<0.05
Food group 6 (dairy products and cheeses)	0.80	0.61, 1.04	0.088	0.80	0.61, 1.04	0.093
Food group 7 (eggs)	1.14	0.82, 1.62	0.445	1.16	0.82, 1.67	0.412
Food group 8 (fish meat)	0.91	0.64, 1.33	0.625	0.98	0.68, 1.45	0.922
Food group 9 (raw and cooked vegetables with leaves)	0.85	0.66, 1.11	0.239	0.86	0.65, 1.14	0.298
Food group 10 (vegetables and legumes)	0.82	0.62, 1.08	0.157	0.85	0.64, 1.13	0.264
Food group 11 (fruit)	0.78	0.60, 1.03	0.065	0.79	0.61, 1.05	0.097
Water	1.13	0.82, 1.59	0.479	1.22	0.88, 1.74	0.247
Carbonated drinks	0.86	0.59, 1.21	0.403	0.97	0.66, 1.38	0.871
Beer	0.68	0.46, 1.00	0.057	0.86	0.56, 1.28	0.457
Wine	1.08	0.86, 1.36	0.518	1.05	0.81, 1.36	0.713

* Corrected for age, sex, and education. Abbreviations: OR: Odds Ratio; CI: Confidence Interval.

**Table 4:** Odds ratio (95% confidence interval) and related p-value for each factor and amnestic MCI group.

	Raw OR	95% CI	*p*	Corrected * OR	95% CI	*p*
Age	1.09	1.04, 1.13	<0.001			
Sex	0.71	0.41, 1.20	0.209			
Education	0.82	0.75, 0.89	<0.001			
Depression level	1.09	1.00, 1.19	<0.05	1.03	0.94, 1.13	0.508
Familiarity with AD	2.08	0.99, 4.05	<0.05	2.50	1.13, 5.18	<0.05
Traumatic brain injuries (TBIs)	1.00	0.29, 2.57	0.998	0.92	0.27, 2.45	0.885
Perceived physical pain	1.38	1.07, 1.76	<0.05	1.12	0.87, 1.46	0.372
Smoking	0.60	0.22, 1.35	0.256	1.22	0.43, 3.03	0.681
Alcohol	0.76	0.46, 1.21	0.263	0.96	0.57, 1.56	0.860
Waist-Hip Ratio (WHR)	0.06	0.01, 0.84	<0.05	0.08	0.01, 1.67	0.110
Body Mass Index (BMI)	0.98	0.92, 1.04	0.584	0.97	0.90, 1.03	0.288
Second language	0.51	0.24, 0.99	0.061	1.33	0.58, 2.84	0.476
Physical activities	0.75	0.59, 0.91	<0.01	0.83	0.66, 1.02	0.096
Cultural activities	0.66	0.46, 0.89	<0.05	0.92	0.64, 1.25	0.610
Intellectual activities	0.73	0.63, 0.83	<0.001	0.80	0.69, 0.93	<0.01
Productive activities	1.03	0.90, 1.20	0.644	1.03	0.89, 1.21	0.687
Social activities	0.88	0.75, 1.04	0.115	0.93	0.78, 1.11	0.402
Quality of life—physical health	0.90	0.83, 0.98	<.01	0.96	0.88, 1.06	0.414
Quality of life—mental health	0.95	0.89, 1.01	0.069	0.96	0.90, 1.02	0.180
Sleep quality	1.37	1.06, 1.78	<0.05	1.25	0.96, 1.63	0.097
Topographical disorientation self-report	0.94	0.87, 1.01	0.081	0.95	0.88, 1.01	0.099
Topographical disorientation egocentric component	1.32	1.17, 1.48	<0.001	1.30	1.14, 1.48	<0.001
Topographical disorientation allocentric component	1.31	1.16, 1.47	<0.001	1.25	1.09, 1.42	<0.001
Topographical disorientation total	0.84	0.78, 0.90	<0.001	1.18	1.09, 1.27	<0.001
Food group 1 (carbohydrates)	1.26	0.85, 1.88	0.252	1.23	0.82, 1.87	0.327
Food group 2 (cured and smoked meats)	1.05	0.76, 1.47	0.757	1.08	0.77, 1.53	0.669
Food group 3 (white meat)	0.90	0.63, 1.33	0.593	0.77	0.52, 1.16	0.204
Food group 4 (red meat)	1.42	0.97, 2.11	0.078	1.34	0.90, 2.01	0.155
Food group 5 (milk)	0.96	0.79, 1.18	0.695	0.85	0.69, 1.05	0.116
Food group 6 (dairy products and cheeses)	0.72	0.52, 0.99	<0.05	0.72	0.52, 0.99	<0.05
Food group 7 (eggs)	1.28	0.83, 2.01	0.275	1.31	0.84, 2.08	0.249
Food group 8 (fish meat)	0.81	0.53, 1.28	0.356	0.86	0.55, 1.87	0.501
Food group 9 (raw and cooked vegetables with leaves)	0.84	0.61, 1.17	0.303	0.85	0.60, 1.20	0.356
Food group 10 (vegetables and legumes)	0.79	0.55, 1.11	0.178	0.82	0.57, 1.16	0.269
Food group 11 (fruit)	0.74	0.55, 1.03	0.057	0.75	0.55, 1.05	0.082
Water	1.19	0.79, 1.85	0.426	1.32	0.87, 2.08	0.204
Carbonated drinks	0.97	0.62, 1.44	0.898	1.12	0.71, 1.69	0.614
Beer	0.76	0.46, 1.21	0.263	0.96	0.57, 1.56	0.860
Wine	1.16	0.87, 1.55	0.324	1.10	0.80, 1.52	0.550

* Corrected for age, sex, and education. Abbreviations: OR: Odds Ratio; CI: Confidence Interval.

**Table 5:** Odds ratio (95% confidence interval) and related p-value for each factor and non-amnestic MCI group.

	Raw OR	95% CI	*p*	Corrected * OR	95% CI	*p*
Age	1.07	1.02, 1.12	<0.05			
Sex	0.67	0.34, 1.30	0.248			
Education	0.86	0.78, 0.94	<0.01			
Depression level	1.09	0.97, 1.22	0.145	1.03	0.90, 1.16	0.664
Familiarity with AD	0.25	0.01, 1.17	0.170	0.27	0.02, 1.29	0.199
Traumatic brain injuries (TBIs)	0.76	0.12, 2.06	0.715	0.68	0.11, 2.36	0.601
Perceived physical pain	0.83	0.61, 1.13	0.216	1.00	0.73, 1.38	0.975
Smoking	1.22	0.61, 2.38	0.571	2.06	0.94, 4–47	0.068
Alcohol	0.57	0.29, 1.04	0.079	0.70	0.35, 1.32	0.286
Waist-Hip Ratio (WHR)	2.42	0.14, 23.83	0.497	0.68	0.35, 74.93	0.151
Body Mass Index (BMI)	0.94	0.89, 0.99	<0.05	0.95	0.90, 1.01	0.085
Second language	0.49	0.18, 1.10	0.110	1.06	0.36, 2.72	0.914
Physical activities	0.89	0.71, 1.08	0.274	0.98	0.77, 1.20	0.822
Cultural activities	0.78	0.52, 1.07	0.150	1.02	0.67, 1.45	0.929
Intellectual activities	0.80	0.68, 0.94	<0.01	0.88	0.74,1.04	0.134
Productive activities	1.01	0.86, 1.21	0.926	1.00	0.84, 1.20	0.961
Social activities	1.16	0.91, 1.54	0.281	1.24	0.96, 1.67	0.121
Quality of life—physical health	0.93	0.84, 1.04	0.195	1.00	0.90, 1.13	0.979
Quality of life—mental health	1.05	0.96, 1.15	0.314	1.06	0.98, 1.16	0.164
Sleep quality	1.11	0.80, 1.53	0.546	1.01	0.73, 1.41	0.935
Topographical disorientation self-report	1.03	0.96, 1.10	0.420	1.03	0.96, 1.10	0.419
Topographical disorientation egocentric component	0.98	0.84, 1.16	0.799	1.02	0.87, 1.22	0.833
Topographical disorientation allocentric component	0.94	0.82, 1.09	0.378	0.93	0.86, 1.16	0.923
Topographical disorientation total	0.97	0.89, 1.06	0.492	1.00	0.91, 1.11	0.952
Food group 1 (carbohydrates)	0.82	0.52, 1.30	0.387	0.78	0.49, 1.27	0.312
Food group 2 (cured and smoked meats)	0.88	0.60, 1.32	0.542	0.91	0.60, 1.38	0.637
Food group 3 (white meat)	1.14	0.71, 1.91	0.598	1.05	0.64, 1.76	0.862
Food group 4 (red meat)	1.18	0.75, 1.91	0.481	1.17	0.74, 1.88	0.508
Food group 5 (milk)	0.91	0.72, 1.15	0.404	0.83	0.65, 1.06	0.120
Food group 6 (dairy products and cheeses)	0.94	0.63, 1.41	0.762	0.93	0.62, 1.39	0.706
Food group 7 (eggs)	0.97	0.59, 1.64	0.911	0.97	0.59, 1.67	0.920
Food group 8 (fish meat)	1.12	0.64, 2.05	0.713	1.21	0.69, 2.22	0.523
Food group 9 (raw and cooked vegetables with leaves)	0.88	0.59, 1.31	0.516	0.88	0.58, 1.32	0.531
Food group 10 (vegetables and legumes)	0.87	0.57, 1.32	0.521	0.90	0.58, 1.37	0.635
Food group 11 (fruit)	0.87	0.58, 1.38	0.510	0.87	0.58, 1.39	0.522
Water	1.04	0.64, 1.78	0.870	1.13	0.70, 1.93	0.636
Carbonated drinks	0.68	0.35, 1.20	0.222	0.75	0.38, 1.32	0.360
Beer	0.57	0.29, 1.04	0.080	0.70	0.35, 1.32	0.285
Wine	0.97	0.67, 1.39	0.856	0.95	0.64, 1.40	0.791

* Corrected for age, sex, and education. Abbreviations: OR: Odds Ratio; CI: Confidence Interval.

## Data Availability

The data presented in this study are available on request from the corresponding author. The data are not publicly available due to privacy regulations.

## Supplementary Materials

The following supporting information can be downloaded at: https://www.mdpi.com/article/10.3390/ijerph19053097/s1, Table S1: Mean Scores and Standard Deviations or Frequencies on the Variables Included in the Logistic Regression Models for the Three Elderly Groups, and Statistical Tests for Their Differences.

## References

[1] Petersen R.C., Doody R., Kurz A., Mohs R.C., Morris J.C., Rabins P.V., Ritchie K., Rossor M., Thal L., Winblad B. (2001). Current Concepts in Mild Cognitive Impairment. Arch. Neurol..

[2] Mild Cognitive Impairment as a Diagnostic Entity—Petersen—2004—Journal of Internal Medicine—Wiley Online Library. https://onlinelibrary.wiley.com/doi/full/10.1111/j.1365-2796.2004.01388.x.

[3] Albert M.S., DeKosky S.T., Dickson D., Dubois B., Feldman H.H., Fox N.C., Gamst A., Holtzman D.M., Jagust W.J., Petersen R.C. (2011). The Diagnosis of Mild Cognitive Impairment Due to Alzheimer’s Disease: Recommendations from the National Institute on Aging-Alzheimer’s Association Workgroups on Diagnostic Guidelines for Alzheimer’s Disease. Alzheimer’s Dement..

[4] Mitchell A.J., Shiri-Feshki M. (2009). Rate of Progression of Mild Cognitive Impairment to Dementia—Meta-Analysis of 41 Robust Inception Cohort Studies. Acta Psychiatr. Scand..

[5] Ward A., Arrighi H.M., Michels S., Cedarbaum J.M. (2012). Mild Cognitive Impairment: Disparity of Incidence and Prevalence Estimates. Alzheimer’s Dement..

[6] Alexander M., Perera G., Ford L., Arrighi H.M., Foskett N., Debove C., Novak G., Gordon M.F. (2015). Age-stratified prevalence of mild cognitive impairment and dementia in European populations: A systematic review. J. Alzheimer’s Dis..

[7] Hu C., Yu D., Sun X., Zhang M., Wang L., Qin H. (2017). The Prevalence and Progression of Mild Cognitive Impairment among Clinic and Community Populations: A Systematic Review and Meta-Analysis. Int. Psychogeriatr..

[8] Pessoa R.M.P., Bomfim A.J.L., Ferreira B.L.C., Chagas M.H.N. (2019). Diagnostic Criteria and Prevalence of Mild Cognitive Impairment in Older Adults Living in the Community: A Systematic Review and Meta-Analysis. Arch. Clin. Psychiatry.

[9] Parnetti L., Chipi E., Salvadori N., D’Andrea K., Eusebi P. (2019). Prevalence and Risk of Progression of Preclinical Alzheimer’s Disease Stages: A Systematic Review and Meta-Analysis. Alzheimer’s Res. Ther..

[10] Zhang H., Zhong D., Li J., Liu Y., Jin R. (2020). Epidemiological of mild cognitive impairment in Chinese elderly population: A systematic review. Chin. J. Evid.-Based Med..

[11] Deng Y., Zhao S., Cheng G., Yang J., Li B., Xu K., Xiao P., Li W., Rong S. (2021). The Prevalence of Mild Cognitive Impairment among Chinese People: A Meta-Analysis. Neuroepidemiology.

[12] Lu Y., Liu C., Yu D., Fawkes S., Ma J., Zhang M., Li C. (2021). Prevalence of Mild Cognitive Impairment in Community-Dwelling Chinese Populations Aged over 55 Years: A Meta-Analysis and Systematic Review. BMC Geriatr..

[13] Ribeiro F.S., Teixeira-Santos A.C., Leist A.K. (2021). The Prevalence of Mild Cognitive Impairment in Latin America and the Caribbean: A Systematic Review and Meta-Analysis. Aging Ment. Health.

[14] Prencipe M., Santini M., Casini A.R., Pezzella F.R., Scaldaferri N., Culasso F. (2003). Prevalence of Non-Dementingcognitive Disturbances and Theirassociation with Vascular Risk Factorsin an Elderly Population. J. Neurol..

[15] Solfrizzi V., Panza F., Colacicco A.M., D’Introno A., Capurso C., Torres F., Grigoletto F., Maggi S., Parigi A.D., Reiman E.M. (2004). Vascular Risk Factors, Incidence of MCI, and Rates of Progression to Dementia. Neurology.

[16] Zanetti M., Ballabio C., Abbate C., Cutaia C., Vergani C., Bergamaschini L. (2006). Mild Cognitive Impairment Subtypes and Vascular Dementia in Community-Dwelling Elderly People: A 3-Year Follow-Up Study. J. Am. Geriatr. Soc..

[17] Solfrizzi V., D’Introno A., Colacicco A.M., Capurso C., Parigi A.D., Caselli R.J., Scapicchio P.L., Scafato E., Gandin C., Capurso A. (2007). Incident Occurrence of Depressive Symptoms among Patients with Mild Cognitive Impairment—The Italian Longitudinal Study on Aging. Dement. Geriatr. Cogn. Disord..

[18] Carlo A.D., Lamassa M., Baldereschi M., Inzitari M., Scafato E., Farchi G., Inzitari D. (2007). CIND and MCI in the Italian Elderly: Frequency, Vascular Risk Factors, Progression to Dementia. Neurology.

[19] Ravaglia G., Forti P., Montesi F., Lucicesare A., Pisacane N., Rietti E., Dalmonte E., Bianchin M., Mecocci P. (2008). Mild Cognitive Impairment: Epidemiology and Dementia Risk in an Elderly Italian Population. J. Am. Geriatr. Soc..

[20] Moretti F., De Ronchi D., Palmer K., Forlani C., Morini V., Ferrari B., Dalmonte E., Atti A.R. (2013). Prevalence and Characteristics of Mild Cognitive Impairment in the General Population. Data from an Italian Population-Based Study: The Faenza Project. Aging Ment. Health.

[21] Guaita A., Vaccaro R., Davin A., Colombo M., Vitali S.F., Polito L., Abbondanza S., Valle E., Forloni G., Ferretti V.V. (2015). Influence of Socio-Demographic Features and Apolipoprotein E Epsilon 4 Expression on the Prevalence of Dementia and Cognitive Impairment in a Population of 70–74-Year Olds: The InveCe.Ab Study. Arch. Gerontol. Geriatr..

[22] Limongi F., Siviero P., Noale M., Gesmundo A., Crepaldi G., Maggi S., Dementia Registry Study Group (2017). Prevalence and Conversion to Dementia of Mild Cognitive Impairment in an Elderly Italian Population. Aging Clin. Exp. Res..

[23] Solfrizzi V., Scafato E., Lozupone M., Seripa D., Giannini M., Sardone R., Bonfiglio C., Abbrescia D.I., Galluzzo L., Gandin C. (2017). Additive Role of a Potentially Reversible Cognitive Frailty Model and Inflammatory State on the Risk of Disability: The Italian Longitudinal Study on Aging. Am. J. Geriatr. Psychiatry.

[24] Beydoun M.A., Beydoun H.A., Gamaldo A.A., Teel A., Zonderman A.B., Wang Y. (2014). Epidemiologic Studies of Modifiable Factors Associated with Cognition and Dementia: Systematic Review and Meta-Analysis. BMC Public Health.

[25] Baumgart M., Snyder H.M., Carrillo M.C., Fazio S., Kim H., Johns H. (2015). Summary of the Evidence on Modifiable Risk Factors for Cognitive Decline and Dementia: A Population-Based Perspective. Alzheimer’s Dement..

[26] Xu W., Tan L., Wang H.-F., Jiang T., Tan M.-S., Tan L., Zhao Q.-F., Li J.-Q., Wang J., Yu J.-T. (2015). Meta-Analysis of Modifiable Risk Factors for Alzheimer’s Disease. J. Neurol. Neurosurg. Psychiatry.

[27] Cooper C., Sommerlad A., Lyketsos C.G., Livingston G. (2015). Modifiable Predictors of Dementia in Mild Cognitive Impairment: A Systematic Review and Meta-Analysis. Am. J. Psychiatry.

[28] Aguirre G.K., D’Esposito M. (1999). Topographical Disorientation: A Synthesis and Taxonomy. Brain.

[29] Tabert M.H., Manly J.J., Liu X., Pelton G.H., Rosenblum S., Jacobs M., Zamora D., Goodkind M., Bell K., Stern Y. (2006). Neuropsychological Prediction of Conversion to Alzheimer Disease in Patients With Mild Cognitive Impairment. Arch. Gen. Psychiatry.

[30] Caffò A.O., Lopez A., Spano G., Stasolla F., Serino S., Cipresso P., Riva G., Bosco A. (2020). The Differential Effect of Normal and Pathological Aging on Egocentric and Allocentric Spatial Memory in Navigational and Reaching Space. Neurol. Sci..

[31] Lopez A., Germani A., Tinella L., Caffò A.O., Postma A., Bosco A. (2021). The Road More Travelled: The Differential Effects of Spatial Experience in Young and Elderly Participants. Int. J. Environ. Res. Public Health.

[32] Lopez A., Caffò A.O., Postma A., Bosco A. (2020). How to Separate Coordinate and Categorical Spatial Relation Components in Integrated Spatial Representations: A New Methodology for Analysing Sketch Maps. Scand. J. Psychol..

[33] Portet F., Ousset P.J., Visser P.J., Frisoni G.B., Nobili F., Scheltens P., Vellas B., Touchon J. (2006). Disease (EADC), the M.W.G. of the E.C. on A. Mild Cognitive Impairment (MCI) in Medical Practice: A Critical Review of the Concept and New Diagnostic Procedure. Report of the MCI Working Group of the European Consortium on Alzheimer’s Disease. J. Neurol. Neurosurg. Psychiatry.

[34] Bosco A., Spano G., Caffò A.O., Lopez A., Grattagliano I., Saracino G., Pinto K., Hoogeveen F., Lancioni G.E. (2017). Italians Do It Worse. Montreal Cognitive Assessment (MoCA) Optimal Cut-off Scores for People with Probable Alzheimer’s Disease and with Probable Cognitive Impairment. Aging Clin. Exp. Res..

[35] Tinella L., Lopez A., Caffò A.O., Nardulli F., Grattagliano I., Bosco A. (2021). Cognitive Efficiency and Fitness-to-Drive along the Lifespan: The Mediation Effect of Visuospatial Transformations. Brain Sci..

[36] Assessing Self-maintenance: Activities of Daily Living, Mobility, and Instrumental Activities of Daily Living—KATZ—1983. *J. Am. Geriatr. Soc.* Wiley Online Library. https://agsjournals.onlinelibrary.wiley.com/doi/abs/10.1111/j.1532-5415.1983.tb03391.x.

[37] Hopman-Rock M., van Hirtum H., de Vreede P., Freiberger E. (2019). Activities of Daily Living in Older Community-Dwelling Persons: A Systematic Review of Psychometric Properties of Instruments. Aging Clin. Exp. Res..

[38] Quesada J.J., Ferrucci L., Calvani D., Valente C., Salani B., Bavazzano A. (1997). Formal Education as an Effect Modifier of the Relationship between Mini-Mental State Examination Score and IADLs Disability in the Older Population. Aging Clin. Exp. Res..

[39] Brink T.L., Yesavage J.A., Lum O., Heersema P.H., Adey M., Rose T.L. (1982). Screening Tests for Geriatric Depression. Clin. Gerontol..

[40] Parmelee P.A., Lawton M.P., Katz I.R. (1989). Psychometric Properties of the Geriatric Depression Scale among the Institutionalized Aged. Psychol. Assess. J. Consult. Clin. Psychol..

[41] Greenberg S.A. (2007). How To try this: The Geriatric Depression ScaleShort Form. Am. J. Nurs..

[42] Spano G., Caffò A., Bosco A. (2018). Cognitive Functioning, Subjective Memory Complaints and Risky Behaviour Predict Minor Home Injuries in Elderly. Aging Clin. Exp. Res..

[43] Spano G., Caffò A.O., Lanciano T., Curci A., Bosco A. (2020). Visuospatial/Executive Abilities and Mood Affect the Reliability of a Subjective Memory Complaints Measure. Aging Clin. Exp. Res..

[44] Youn J.C., Kim K.W., Lee D.Y., Jhoo J.H., Lee S.B., Park J.H., Choi E.A., Choe J.Y., Jeong J.W., Choo I.H. (2009). Development of the Subjective Memory Complaints Questionnaire. Dement. Geriatr. Cogn. Disord..

[45] Yim S.J., Yi D., Byun M.S., Choe Y.M., Choi H.J., Baek H., Sohn B.K., Kim J.W., Kim E.-J., Lee D.Y. (2017). Screening Ability of Subjective Memory Complaints, Informant-Reports for Cognitive Decline, and Their Combination in Memory Clinic Setting. Psychiatry Investig..

[46] Bean J., Kreutzer J.S., DeLuca J., Caplan B. (2011). Rey Auditory Verbal Learning Test, Rey AVLT. Encyclopedia of Clinical Neuropsychology.

[47] Carlesimo G.A., Caltagirone C., Gainotti G., Fadda L., Gallassi R., Lorusso S., Marfia G., Marra C., Nocentini U., Parnetti L. (1996). The Mental Deterioration Battery: Normative Data, Diagnostic Reliability and Qualitative Analyses of Cognitive Impairment. ENE.

[48] Appollonio I., Leone M., Isella V., Piamarta F., Consoli T., Villa M.L., Forapani E., Russo A., Nichelli P. (2005). The Frontal Assessment Battery (FAB): Normative Values in an Italian Population Sample. Neurol. Sci..

[49] Spinnler H., Tognoni G. (1987). Standardizzazione e Taratura Italiana di Test Neuropsicologici: Gruppo Italiano per lo Studio Neuropsicologico dell’Invecchiamento.

[50] Caffarra P., Gardini S., Zonato F., Concari L., Dieci F., Copelli S., Freedman M., Stracciari A., Venneri A. (2011). Italian Norms for the Freedman Version of the Clock Drawing Test. J. Clin. Exp. Neuropsychol..

[51] Yener G., Tunçay N., Kayserili G., Erhan E., Akdede B., Zorlu Y. (2008). Validation and Reliability of The Frontal Assesment Battery (FAB) in Turkish. Front. Hum. Neurosci..

[52] Can S.S., Gencay-Can A., Gunendi Z. (2012). Validity and Reliability of the Clock Drawing Test as a Screening Tool for Cognitive Impairment in Patients with Fibromyalgia. Compr. Psychiatry.

[53] Pai M.-C., Lee C.-C., Yang Y.-C., Lee Y.-T., Chen K.-C., Lin S.-H., Jheng S.-S., Sun P.-W., Cheng P.-J. (2012). Development of a Questionnaire on Everyday Navigational Ability to Assess Topographical Disorientation in Alzheimer’s Disease. Am. J. Alzheimer’s Dis. Dement..

[54] Ruggiero G., Iachini T., Ruotolo F., Senese V.P. (2009). Spatial Memory: The Role of Egocentric and Allocentric Frames Of Reference.

[55] R: The R Project for Statistical Computing. https://www.r-project.org/.

[56] West S.G., Finch J.F., Curran P.J. (1995). Structural Equation Models with Nonnormal Variables: Problems and Remedies. Structural Equation Modeling: Concepts, Issues, and Applications.

[57] Tognoni G., Ceravolo R., Nucciarone B., Bianchi F., Dell’Agnello G., Ghicopulos I., Siciliano G., Murri L. (2005). From Mild Cognitive Impairment to Dementia: A Prevalence Study in a District of Tuscany, Italy. Acta Neurol. Scand..

[58] Caamaño-Isorna F., Corral M., Montes-Martínez A., Takkouche B. (2006). Education and Dementia: A Meta-Analytic Study. Neuroepidemiology.

[59] Sharp E.S., Gatz M. (2011). The Relationship between Education and Dementia An Updated Systematic Review. Alzheimer Dis. Assoc. Disord..

[60] Maccora J., Peters R., Anstey K.J. (2020). What Does (Low) Education Mean in Terms of Dementia Risk? A Systematic Review and Meta-Analysis Highlighting Inconsistency in Measuring and Operationalising Education. SSM-Popul. Health.

[61] Caffò A.O., Lopez A., Spano G., Saracino G., Stasolla F., Ciriello G., Grattagliano I., Lancioni G.E., Bosco A. (2016). The Role of Pre-Morbid Intelligence and Cognitive Reserve in Predicting Cognitive Efficiency in a Sample of Italian Elderly. Aging Clin. Exp. Res..

[62] What Is Cognitive Reserve? Theory and Research Application of the Reserve Concept. *J. Int. Neuropsychol. Soc.* Cambridge Core. https://www.cambridge.org/core/journals/journal-of-the-international-neuropsychological-society/article/abs/what-is-cognitive-reserve-ory-and-research-application-of-the-reserve-concept/B6524DF8FC814A462004141F7B19BCF4.

[63] Valenzuela M.J., Sachdev P. (2006). Brain Reserve and Dementia: A Systematic Review. Psychol. Med..

[64] Pai M.-C., Jacobs W.J. (2004). Topographical Disorientation in Community-Residing Patients with Alzheimer’s Disease. Int. J. Geriatr. Psychiatry.

[65] Hort J., Laczó J., Vyhnálek M., Bojar M., Bureš J., Vlček K. (2007). Spatial Navigation Deficit in Amnestic Mild Cognitive Impairment. Proc. Natl. Acad. Sci. USA.

[66] Weniger G., Ruhleder M., Lange C., Wolf S., Irle E. (2011). Egocentric and Allocentric Memory as Assessed by Virtual Reality in Individuals with Amnestic Mild Cognitive Impairment. Neuropsychologia.

[67] Rusconi M.L., Suardi A., Zanetti M., Rozzini L. (2015). Spatial Navigation in Elderly Healthy Subjects, Amnestic and Non Amnestic MCI Patients. J. Neurol. Sci..

[68] Chan J.Y.C., Yiu K.K.L., Kwok T.C.Y., Wong S.Y.S., Tsoi K.K.F. (2019). Depression and Antidepressants as Potential Risk Factors in Dementia: A Systematic Review and Meta-Analysis of 18 Longitudinal Studies. J. Am. Med. Dir. Assoc..

[69] Lautenschlager N.T., Cox K.L., Ellis K.A. (2019). Physical Activity for Cognitive Health: What Advice Can We Give to Older Adults with Subjective Cognitive Decline and Mild Cognitive Impairment?. Dialogues Clin. Neurosci..

[70] Wang Z., Hou J., Shi Y., Tan Q., Peng L., Deng Z., Wang Z., Guo Z. (2020). Influence of Lifestyles on Mild Cognitive Impairment: A Decision Tree Model Study. Clin. Interv. Aging.

[71] Feng L., Cheah I.K.-M., Ng M.M.-X., Li J., Chan S.M., Lim S.L., Mahendran R., Kua E.-H., Halliwell B. (2019). The Association between Mushroom Consumption and Mild Cognitive Impairment: A Community-Based Cross-Sectional Study in Singapore. J. Alzheimer’s Dis..

[72] Assaf G., El Khoury J., Jawhar S., Rahme D. (2021). Mild Cognitive Impairment and Modifiable Risk Factors among Lebanese Older Adults in Primary Care. Asian J. Psychiatry.

[73] Boo Y.Y., Jutila O.-E., Cupp M.A., Manikam L., Cho S.-I. (2021). The Identification of Established Modifiable Mid-Life Risk Factors for Cardiovascular Disease Which Contribute to Cognitive Decline: Korean Longitudinal Study of Aging (KLoSA). Aging Clin. Exp. Res..

[74] Busse A., Angermeyer M.C., Riedel-Heller S.G. (2006). Progression of Mild Cognitive Impairment to Dementia: A Challenge to Current Thinking. Br. J. Psychiatry.

