# Supplementary material for: The Prevalence of Amnestic and Non-Amnestic Mild Cognitive Impairment and Its Association with Different Lifestyle Factors in a South Italian Elderly Population
Source: Int J Environ Res Public Health. 2022 Mar 6;19(5):3097. doi: 10.3390/ijerph19053097 (PMC8910691; doi:10.3390/ijerph19053097)
Supplement: Supplementary file 1 [file ijerph-19-03097-s001.zip › ijerph-1571929-supplementary.pdf]

Table S1. Mean Scores and Standard Deviations or Frequencies on the Variables Included in the Logistic Regression Models for the Three Elderly Groups, and Statistical Tests for Their Differences.

	Healthy Elderly (N = 738)	amnestic MCI (N = 62)	non amnestic MCI (N = 39)	F o χ2	p	Post hoc
Age	72.36 ± 6.44	75.87 ± 6.56	75.13 ± 6.68	11.16	<.001	HE < aMCI = naMCI
Sex (F/M)	403/335	39/23	25/14	2.78	.250	
Education	9.44 ± 4.78	6.23 ± 3.39	6.77 ± 4.58	18.38	<.001	HE > aMCI = naMCI
Depression level	2.74 ± 2.58	3.45 ± 3.28	3.37 ± 2.16	2.88	0.057	HE = aMCI = naMCI
Familiarity with AD	630/71	47/11	36/3	6.96	0.031	HE < aMCI = naMCI
Traumatic brain injuries (TBI)	673/49	55/4	36/2	0.134	0.935	HE = aMCI = naMCI
Perceived Physical Pain	4.00 ± 1.01	3.65 ± 1.07	3.79 ± 1.13	3.50	0.036	HE < aMCI = naMCI
Smoking	371/274/91	41/14/6	19/17/2	8.70	0.069	HE < aMCI = naMCI
Alcohol	649/62/12/2	57/3/1	35/1/2	4.23	0.835	HE = aMCI = naMCI
Waist-Hip Ratio (WHR)	0.91 ± 0.12	0.88 ± 0.12	0.93 ± 0.12	2.32	0.107	HE = aMCI = naMCI
Body Mass Index (BMI)	27.1 ± 4.38	26.9 ± 3.92	28.9 ± 5.11	2.26	0.113	HE = aMCI = naMCI
Second language	537/201	52/10	33/6	6.02	0.049	HE < aMCI = naMCI
Physical activities	1.14 ± 1.67	0.48 ± 1.27	0.82 ± 1.54	7.60	0.001	HE < aMCI = naMCI
Cultural activities	0.80 ± 1.10	0.43 ± 0.99	0.54 ± 0.97	4.91	0.010	HE < aMCI = naMCI
Intellectual activities	2.98 ± 1.97	1.67 ± 1.94	2.08 ± 2.11	15.4	<.001	HE < aMCI = naMCI
Productive activities	3.41 ± 1.19	3.52 ± 1.97	3.44 ± 1.90	0.10	0.903	HE = aMCI = naMCI
Social activities	3.38 ± 1.42	3.52 ± 1.49	4.08 ± 1.26	1.99	0.144	HE = aMCI = naMCI
Quality of life – physical health	15.4 ± 2.86	14.4 ± 3.15	14.8 ± 2.81	3.63	0.032	HE < aMCI = naMCI
Quality of life – mental health	21.2 ± 3.94	20.02 ± 5.02	21.8 ± 3.66	1.76	0.179	HE = aMCI = naMCI
Sleep quality	6.73 ± 2.11	7.26 ± 2.09	6.64 ± 2.29	1.89	0.158	HE = aMCI = naMCI
Topographical disorientation self-report	5.25 ± 4.27	4.25 ± 4.39	5.82 ± 4.24	1.86	0.164	HE = aMCI = naMCI
Topographical disorientation egocentric component	5.93 ± 2.00	4.56 ± 2.02	5.84 ± 1.57	11.00	<.001	HE < aMCI = naMCI

Topographical disorientation allocentric component	5.38 ± 2.19	3.96 ± 1.92	5.05 ± 2.45	12.9	<.001	HE < aMCI = naMCI
Topographical disorientation total	11.3 ± 3.59	8.52 ± 3.29	10.9 ± 3.36	17.00	<.001	HE < aMCI < naMCI
Food group 1 (carbohydrates)	2.99 ± 0.68	3.10 ± 0.62	2.90 ± 0.68	1.21	0.304	HE = aMCI = naMCI
Food group 2 (cured and smoked meats)	1.54 ± 0.80	1.58 ± 0.93	1.46 ± 0.79	0.24	0.789	HE = aMCI = naMCI
Food group 3 (white meat)	1.74 ± 0.68	1.69 ± 0.67	1.79 ± 0.52	0.39	0.676	HE = aMCI = naMCI
Food group 4 (red meat)	1.46 ± 0.71	1.62 ± 0.71	1.54 ± 0.76	1.69	0.192	HE = aMCI = naMCI
Food group 5 (milk)	2.23 ± 1.31	2.16 ± 1.25	2.05 ± 1.39	0.38	0.689	HE = aMCI = naMCI
Food group 6 (dairy products and cheeses)	2.01 ± 0.79	1.80 ± 0.75	1.97 ± 0.74	2.20	0.119	HE = aMCI = naMCI
Food group 7 (eggs)	1.52 ± 0.63	1.62 ± 0.59	1.51 ± 0.60	0.69	0.503	HE = aMCI = naMCI
Food group 8 (fish meat)	1.71 ± 0.57	1.64 ± 0.55	1.74 ± 0.44	0.59	0.553	HE = aMCI = naMCI
Food group 9 (raw and cooked vegetables with leaves)	2.47 ± 0.81	2.36 ± 0.73	2.38 ± 0.82	0.79	0.475	HE = aMCI = naMCI
Food group 10 (vegetables and legumes)	2.47 ± 0.78	2.33 ± 0.72	2.38 ± 7.47	1.17	0.318	HE = aMCI = naMCI
Food group 11 (fruit)	3.51 ± 0.69	3.33 ± 0.93	3.44 ± 0.64	1.33	0.272	HE = aMCI = naMCI
Water	3.46 ± 0.65	3.52 ± 0.59	3.47 ± 0.69	0.37	0.691	HE = aMCI = naMCI
Carbonated drinks	0.44 ± 0.64	0.43 ± 0.59	0.32 ± 0.47	1.28	0.284	HE = aMCI = naMCI
Beer	0.54 ± 0.58	0.45 ± 0.62	0.37 ± 0.54	2.17	0.122	HE = aMCI = naMCI
Wine	1.08 ± 0.91	1.21 ± 0.89	1.05 ± 0.89	0.52	0.590	HE = aMCI = naMCI

Abbreviations: aMCI: amnestic MCI; naMCI: non amnestic MCI; F: Female; M: Male.
